# Diffuse Hepatic Hemangiomatosis with Giant Cavernous Hemangioma in an Adult

**DOI:** 10.5334/jbsr.2806

**Published:** 2022-04-25

**Authors:** Olaia Chalh, Nabil Moatassim Billah, Ittimad Nassar

**Affiliations:** 1 Ibn Sina Teaching hospital, MA

**Keywords:** giant hemangioma, diffuse hemangiomatosis, liver, MRI

## Abstract

**Teaching Point:** Although frequent, the coexistence of diffuse hepatic hemangiomatosis (DHH) with giant cavernous hemangioma (GCH) should be mentioned clearly and communicated to the referring physician for an appropriate management.

## Case History

**Figure 1:**
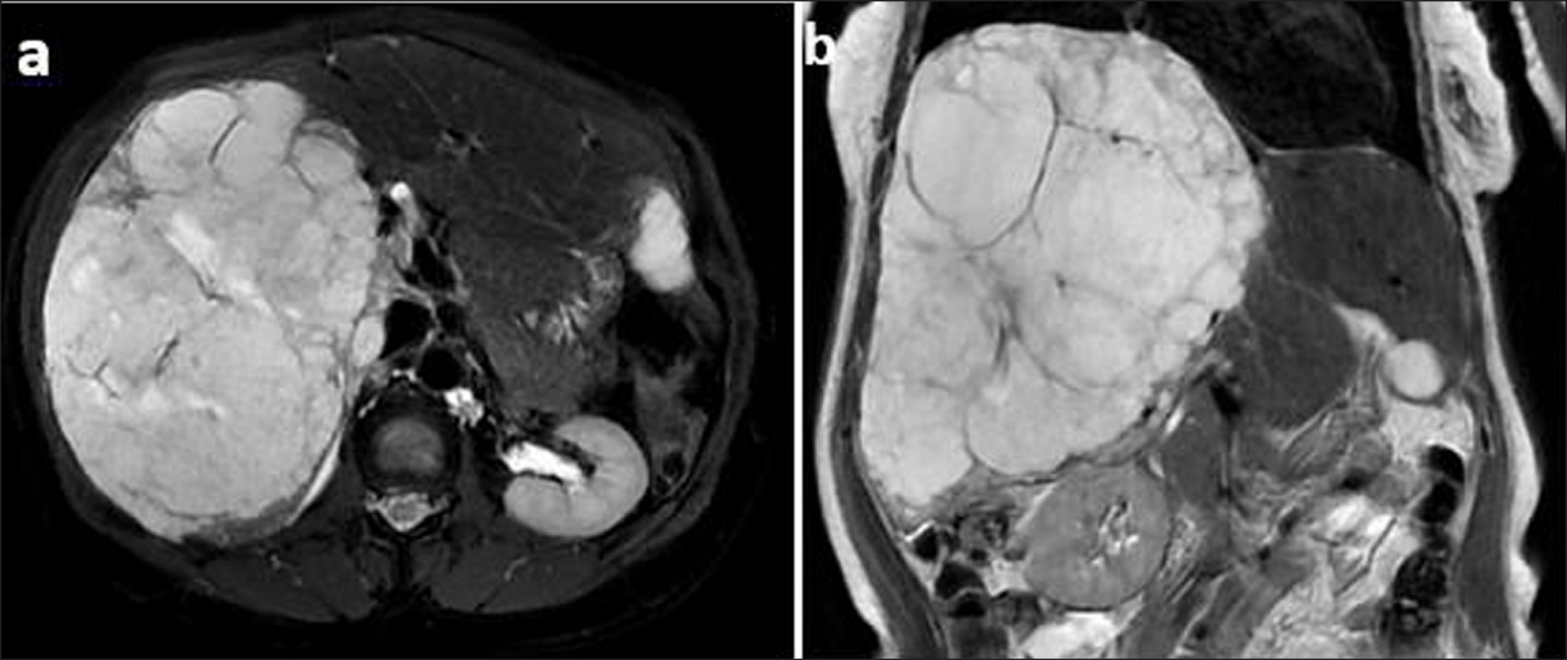


**Figure 2:**
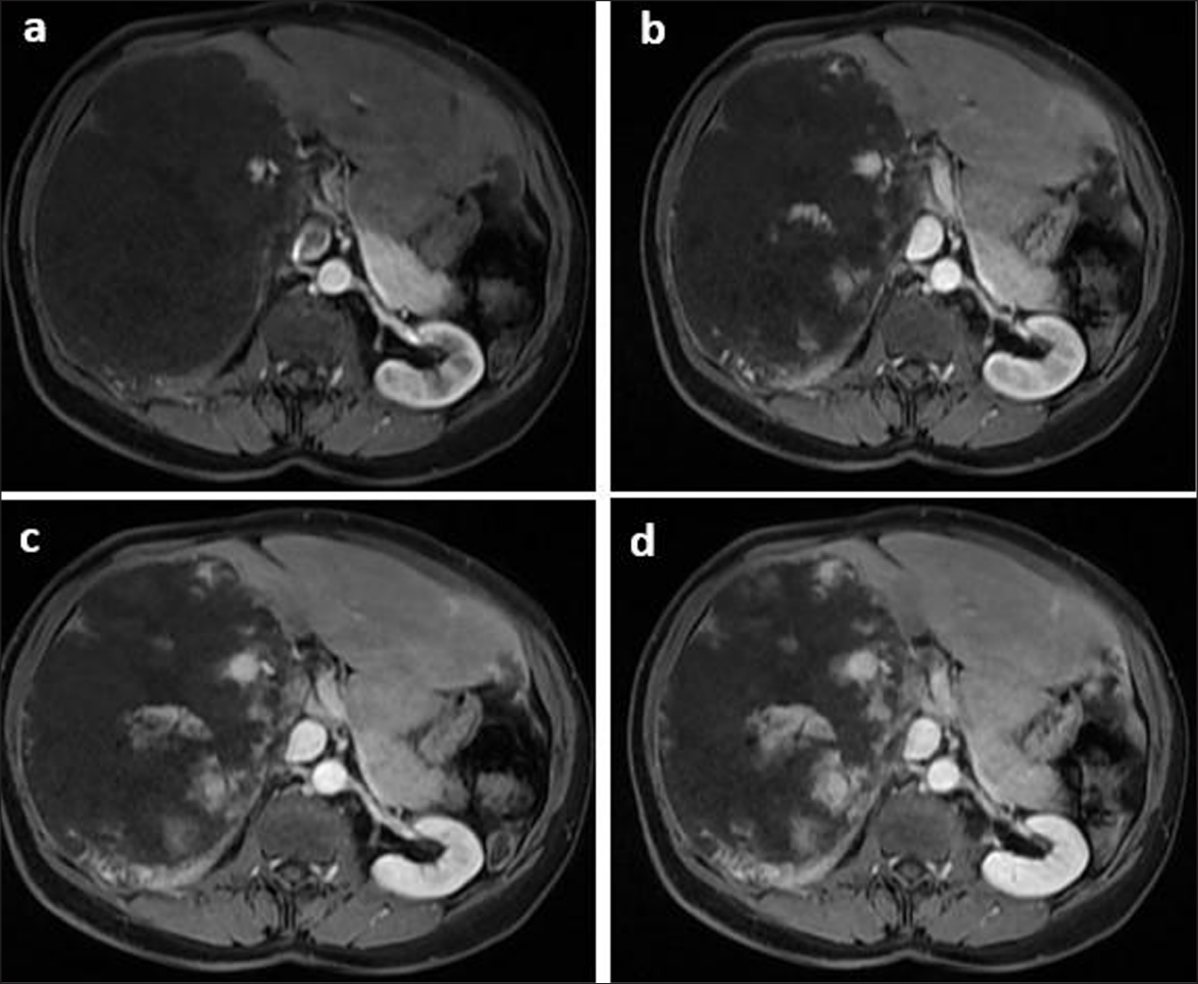


**Figure 3:**
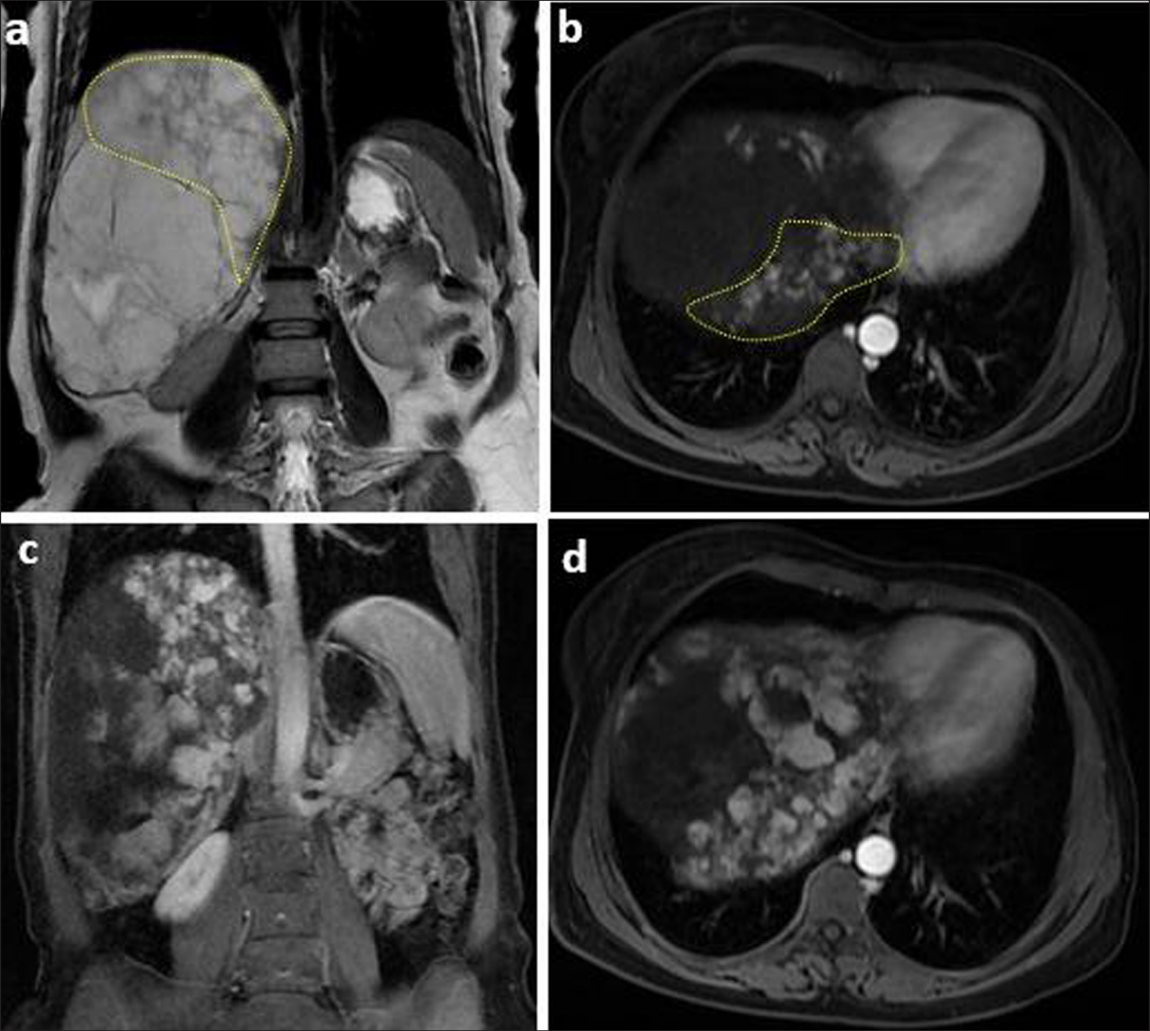


A 56-years-old female with no past medical history presented with suspected atypical hemangioma of the liver causing chronic abdominal discomfort. Laboratory tests were normal. At Magnetic resonance imaging (MRI), axial and coronal single-shot fast spin-echo T2-weighted images revealed a 19 × 18 × 16 cm well-defined mass with lobulated margins replacing the right liver. The mass was hyperintense with central loculations of higher signal intensity and numerous thin hypointense septa (***Figure 1a, b***). Dynamic gadolinium-enhanced images in the axial plane showed peripheral nodular enhancement in the arterial phase and partial centripetal filling in the delayed phases, which is characteristic of GCH (***Figure 2a–d***). Significant mass effect on adjacent organs and vasculature was observed, However, there were no signs of rupture or hemorrhage. In addition, an ill-defined area of confluent hyperintense nodules separated by hypointense septations was seen in posterior segments adjacent to GCH. This area showed heterogeneous discontinuous nodular enhancement in arterial phase which gradually increased in delayed phase (***Figure 3a–d***). The left lobe has expanded without any lesion. The diagnosis of GCH with adjacent DHH was made and the patient was referred to surgical department for therapeutic management.

## Commentary

Giant cavernous hemangioma (GCH) is an atypical form of hepatic hemangioma characterized by a large size, exceeding 5 cm. Symptomatic lesions require surgical management, which depends not only on the tumor’s size but also on the quality of residual liver parenchyma [1]. Diffuse hepatic hemangiomatosis (DHH) is a rare condition of unknown cause characterized by the replacement of the hepatic parenchyma with hemangiomatous lesions. The coexistence of GCH and DHH is not rare [1].

Unlike GHC, which have smooth well-defined margins with classic findings on imaging modalities, DHH appears as an ill-defined area often adjacent to GCH, forming by scattered or confluent innumerable small nodules hypodense on unenhanced CT, hypointense on T1-weighted and hyperintense on T2-weighted images [1]. Two patterns of enhancement may be identified on dynamic contrast phases. A scattered pattern shows heterogeneous hypervascularity during the arterial phase, which gradually becomes homogeneous during delayed imaging, while coalescent nodular types show peripheral centripetal filling in of nodules on a background of heterogeneously enhancing tissue [1]. On ultrasound, DHH appears as homogeneous hyperechoic areas with poorly defined margins or heterogeneous with multiple coalescent small hypoechoic nodules on a hyperechoic background [1].

Management strategies of symptomatic GCH depend on the presence and extent of DHH. These cases can be difficult to enucleate and are better managed by extensive lobectomy or hepatic transplantation. Thus, imaging assessment of residual hepatic parenchyma is highly recommended [1].
